# Convergent Neural Correlates of Empathy and Anxiety During Socioemotional Processing

**DOI:** 10.3389/fnhum.2019.00094

**Published:** 2019-03-19

**Authors:** Lindsay K. Knight, Teodora Stoica, Nicholas D. Fogleman, Brendan E. Depue

**Affiliations:** ^1^Interdisciplinary Program in Translational Neuroscience (IPTN), University of Louisville, Louisville, KY, United States; ^2^Department of Psychological and Brain Sciences, University of Louisville, Louisville, KY, United States; ^3^Department of Anatomical Sciences and Neurobiology, University of Louisville, Louisville, KY, United States

**Keywords:** empathy, anxiety, worry, rumination, social cognition, fMRI

## Abstract

Empathy is characterized by the ability to understand and share an emotional experience with another person and is closely tied to compassion and concern for others. Consequently, this increased emotional awareness and sensitivity may also be related to increased anxiety. Taken from another perspective, higher general anxiety may translate into increased concern for others, or concern for how one’s actions might affect others, and therefore may be linked to increased empathy. Furthermore, self-reflection is positively related to perspective-taking and empathic concern, while rumination is closely tied to anxiety, thus providing an additional connecting point between empathy and anxiety through enhanced internally generated thought. While previous literature suggests a relationship between empathy and anxiety, this has yet to be empirically studied using neuroimaging tools aimed at investigating the underlying neural correlates that may support these convergent responses. We therefore conducted an functional magnetic resonance imaging (fMRI) study (*N* = 49) in which participants viewed fearful and neutral human faces and rated how the faces made them feel, to promote introspection. Participants also completed questionnaires assessing empathy Toronto Empathy Questionnaire (TEQ), trait anxiety State Trait Anxiety Inventory (STAI), worry Penn State Worry Questionnaire (PSWQ) and rumination Ruminative Responses Scale (RRS). Behaviorally, empathy positively correlated with worry, worry and rumination positively correlated with anxiety, and significant indirect relationships were found between empathy and anxiety through worry and rumination. Using the neuroimaging face processing task as a backdrop on which the neurobiological mechanisms of empathy and anxiety may interact, regressions of questionnaires with brain activations revealed that empathy related to activation in the temporoparietal junction (TPJ), anxiety related to bilateral insula activation, and worry related to medial prefrontal cortex (mPFC) activation, while rumination showed increased engagement of all three aforementioned regions. Functional connectivity (FC) analyses showed increased communication between the left amygdala and insula related to higher empathy, worry and rumination. Finally, whole-brain analysis using median split groups from questionnaires revealed that the lower halves of anxiety, worry and rumination exhibited increased activation in top-down attentional networks. In sum, empathy, worry and rumination related to enhanced bottom-up processing, while worry, rumination and anxiety exhibited decreased top-down attentional control, suggesting an indirect relationship between empathy and anxiety through the ruminative tendencies of worry.

## Introduction

### Empathy

Empathy is characterized by the ability to understand and share an emotional experience with another person. This socioemotional response induced by perceiving another person’s affective state is a fundamental component of social interactions and is thought to aid in both moral development and prosocial behavior (Decety et al., 2015). Most literature suggests that empathy encompasses two interrelated components: affective empathy and cognitive empathy (Davis et al., 1994). Affective empathy, also termed empathetic arousal, is the automatic process of vicariously experiencing the emotional state of another person (I feel what you feel). In comparison, cognitive empathy is related to perspective-taking, or the ability to adopt another’s psychological point-of-view, also referred to as mentalizing (I understand what you feel; Davis et al., 1994). While affective empathy is believed to be more innate, fostering care and concern for others, cognitive empathy involves a deliberate understanding of another person’s viewpoint and is particularly important for social competence and reasoning (Decety et al., 2015), as the inability to understand another person’s beliefs and actions may interfere with appropriate social responses (Ickes, 1997; Shamay-Tsoory et al., 2003; Tibi-Elhanany and Shamay-Tsoory, 2011).

### Relation Between Empathy and Anxiety

Given that empathy is closely tied to compassion and concern for others, the increased emotional awareness and social sensitivity in empathy may consequently be associated with anxiety, particularly when empathizing with individuals in distressing situations. Conversely, increased anxiety may translate into increased worry and concern for others, or concern for how one’s actions might affect others, and therefore may be linked to increased empathy. Gaining insight from clinical anxiety, individuals with social anxiety disorder tend to be hyper-self-aware and extremely attentive to social signals due to their intense fear of being negatively evaluated by peers (Tibi-Elhanany and Shamay-Tsoory, 2011). This understanding may help explain why anxious individuals have a propensity to over-interpret the implied threat in others’ facial expressions (Horley et al., 2004), but moreover, these internal and external attentional biases found in anxiety may also be related to enhanced socioemotional processing abilities (Tibi-Elhanany and Shamay-Tsoory, 2011). Indeed, it has been suggested that increased sensitivity to subtle social cues is essential to make mentalizing judgments (Harkness et al., 2005).

Beyond commonalities of enhanced emotional and social sensitivity, empathy and anxiety may additionally be related through increased internally generated thought. Research indicates that enhanced self-reflection is positively correlated with perspective-taking and empathic concern (Joireman et al., 2002) and may even enhance sensitivity leading to more accurate judgments about other’s mental states (Dimaggio et al., 2008). Building on this notion, neuroimaging research provides additional evidence that when predicting emotional responses of another person, greater recruitment of emotion-related and mentalizing regions positively correlates with self-report empathy (Hooker et al., 2008). Similarly, internally generated thought is believed to be a key constituent in anxiety. Meta-analyses show associations between rumination and anxiety, with the strongest links being attributed to brooding and emotion-driven rumination (Olatunji et al., 2013). In fact, rumination has been shown to mediate the longitudinal relationship between life stress and symptoms of anxiety in both adolescents and adults (McLaughlin and Nolen-Hoeksema, 2011). Neuroimaging investigations reveal that rumination reliably engages the cortical midline, especially the more anterior portion, such that increased engagement of the medial prefrontal cortex (mPFC) was found when healthy subjects were instructed to adopt a ruminative thinking style in an autobiographical memory task (Kross et al., 2009; Nejad et al., 2013). Aberrant and sustained recruitment of the amygdala has also been implicated in rumination. During one self-referential processing task in depressed patients, the amygdala was found to exhibit more sustained responses to emotional relative to neutral stimuli, with degree of sustained amygdala activation being positively correlated with rumination scores (Siegle et al., 2002). Together, this suggests that empathy and anxiety may be linked not only through a sensitivity to social and emotional information, but also in a shared propensity to continue to process emotional information through reflection and rumination.

Although the literature is scant, previous research provides some evidence for a direct relation between empathy and anxiety. Using film clips depicting victims facing threats, one recent study demonstrated that trait empathy is associated with greater self-reported vicarious anxiety when observing victims, and a follow-up study extended these findings by manipulating levels of state empathy to establish a causal relationship between empathy and vicarious anxiety (Shu et al., 2017). Furthermore, previous studies have documented that individuals who experience emotion mirroring intensely are more prone to personal distress (e.g., anxiety or discomfort; Lamm et al., 2007). Investigations in the clinical realm have reported similar relationships. A recent study on inpatient adolescents found that measures of affective empathy are positively related to all anxiety dimensions (Gambin and Sharp, 2018). Likewise, Tibi-Elhanany and Shamay-Tsoory (2011) found that individuals with social anxiety disorder demonstrate unique socio-cognitive abilities, such that measures of social anxiety and general anxiety positively associated with affective and cognitive empathy, respectively.

### Neuroimaging of Empathy and Anxiety

To date, neuroimaging studies have only separately investigated empathy and anxiety. In regard to empathy, affective and cognitive empathy involve interacting and partially overlapping neural bases (Fan et al., 2011). Because affective empathy has been linked to automaticity relative to its cognitive component, it primarily elicits activations from regions implicated in rapid and prioritized processing of emotion signals, including: the amygdala, hypothalamus, orbitofrontal cortex (OFC) and anterior insula (Decety et al., 2013). By comparison, cognitive empathy, which shares similar neural networks with perspective-taking and mentalizing (Pardini and Nichelli, 2009), additionally involves the superior temporal sulcus (STS), temporoparietal junction (TPJ), fusiform gyrus (FG), and (mPFC; Saxe, 2006). The involvement of STS and TPJ, areas posited to contain mirror neurons (Iriki, 2006), suggests these areas are specifically activated during the conscious determination of intent. Lesion studies and recent clinical work support the involvement of aforementioned regions in relation to empathic responses. In patients with frontotemporal dementia, reduction of gray matter in the amygdala, insula and TPJ were associated with deficits in attribution of mental states (i.e., mentalizing), specifically in the emotional realm (Cerami et al., 2014). Furthermore, lesions in the amygdala and insula have been found to specifically be associated with deficits in affective empathy (Leigh et al., 2013), while patients with mPFC damage show consistent and selective deficits in cognitive empathy but intact emotion recognition and affective empathy (Shamay-Tsoory et al., 2003). For the purposes of this study, however, we did not aim to further disentangle the neural differences between cognitive and affective empathy, but instead applied this knowledge to more precisely investigate the links between empathy and anxiety.

In regard to anxiety, neuroimaging investigations demonstrate that anxiety reliably elicits activations in the amygdala and insula, particularly in relation to negative emotional responses (Etkin and Wager, 2007; Evans et al., 2008). While the amygdala is central to threat detection, orchestrating behavioral and physiological responses, the insula has been implicated in interoceptive awareness and may be particularly important for sensing perturbations in physiological state when experiencing anxiety (LeDoux and Pine, 2016). Patients with social anxiety disorder, PTSD and specific phobia consistently show greater activations than matched control subjects in these structures, however, similar patterns have been observed during fear conditioning in healthy subjects (Etkin and Wager, 2007). Extensive work additionally highlights the role of the hippocampus in anxiety due to its importance in contextual processing, as well as the mPFC, which provides top-down regulatory control to the amygdala, helping to modulate behavior in light of complex environmental information (Shin and Liberzon, 2010; Duval et al., 2015). Interestingly, many of the regions involved in anxiety are also implicated in empathy, namely the amygdala, insula and mPFC. This evidence suggests empathy and anxiety may modulate processing in similar underlying neural networks, as both constructs are associated with regions involved in processing salient stimuli, subjective emotional experience, and understanding complex social interactions.

Therefore, given evidence of a relation between empathy and anxiety, the current study aimed to investigate the underlying convergent neural correlates using a socially relevant task shown to engage cognitive and emotion processes through face processing, introspection and self-report rating of emotional state. This task was then used as a backdrop to explore how differences in trait empathy and anxiety may modulate processing within a socioemotional processing network and to assess points of convergence between empathy and anxiety. The following hypotheses were posited: (1) behaviorally, higher empathy would be related to higher trait anxiety; (2) increased trait anxiety and empathy would share neural substrates in emotional processing structures (e.g., amygdala and insula); while empathy will additionally relate to regions supporting social cognition (e.g., TPJ); and (3) both empathy and anxiety would exhibit increased functional connectivity (FC) *between* regions supporting emotional processing and social cognition (e.g., amygdala to TPJ), thus demonstrating the two constructs are linked within a socioemotional network.

## Materials and Methods

### Participants

Fifty-five healthy young adults were recruited for the present study. All participants were right handed, with normal or corrected-to-normal vision and hearing, and had no disclosed history of neurological or psychiatric disorders. Participants were recruited through on-campus flyers and an online research participation system (SONA Systems), and were compensated for their time. This study was carried out in accordance with the recommendations of the University of Louisville’s Institutional Review Board. All subjects gave written informed consent in accordance with the Declaration of Helsinki. The protocol was approved by the University of Louisville’s Institutional Review Board. A total of six participants were excluded from analyses due to incomplete behavioral data (*N* = 1), incomplete magnetic resonance imaging (MRI) data (*N* = 4), and disclosed psychiatric diagnosis following consent (*N* = 1), leaving 49 participants in the final sample (28 males, 21 females; Ages 18–33; *M* age = 22.00, SD = 4.04).

### Procedure

The present study consisted of multiple visits. On the first visit, participants completed the functional MRI (fMRI) portion of the study at the University of Louisville, School of Medicine. At this time, participants signed consent forms, were briefed on MRI protocol, read through task instructions and completed the Face Processing Task. Within the next 72 h, participants visited the laboratory on the main campus to complete a variety of self-report questionnaires measuring personality traits of empathy and anxiety, along with measurements assessing internally generated thought (i.e., worry and rumination): Toronto Empathy Questionnaire (TEQ; Spreng et al., 2009), State-Trait Anxiety Inventory (STAI; Spielberger et al., 1970), Penn State Worry Questionnaire (PSWQ; Meyer et al., 1990), and Ruminative Responses Scale (RRS; Treynor et al., 2003). The TEQ was chosen as the measure for empathy because it possesses a robust single factor structure and is thought to measure empathy at the broadest level (i.e., capturing both affective and cognitive empathy), and has convergent validity with existing self-report scales and behavioral measures of affective and cognitive empathy (Spreng et al., 2009). The RRS consists of 22 items, comprising three subscales: (1) reflection—turning inward to engage in cognitive problem solving; (2) brooding—comparing one’s current situation with some unachieved standard; and (3) depressive Rumination. Subscale totals of the RRS can be individually utilized, or all items can be summed together for a composite total rumination score, indicative of one’s propensity to engage in repetitive and passive self-focused attention (Treynor et al., 2003). Since all questionnaires were administered one to 3 days following fMRI task scanning, only trait anxiety scores from the STAI were used in analyses. All questionnaires are known to be psychometrically sound, with high internal consistency and test-retest reliability (Meyer et al., 1990; Barnes et al., 2002; Treynor et al., 2003; Spreng et al., 2009).

#### Task Design

**Figure 1:**
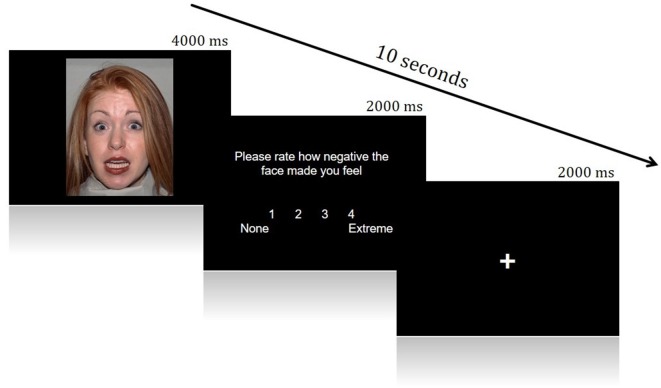
Example of a single trial from the Face Processing Task.

A Face Processing Task constructed from Fearful and Neutral human faces (male and female) shown to reliably elicit activation from brain regions in a canonical socioemotional network was utilized. The Face Processing task consisted of two conditions: Fearful and Neutral. Images were acquired from the Eckman Face Database. During scanning, visual stimuli were displayed through ePrime onto an *in vivo* Esys LCD TV monitor at the back of the scanner bore, which was viewed by participants through a mirror on the head-coil. This task employed an event-related design, during which face stimuli were presented for 4 s in a pseudo-random order (Fear trials *N* = 30, Neutral trials *N* = 12). After presentation of each face, participants rated the image for how negative it made them feel, using a four-point Likert scale (1 = None, 4 = Extremely negative; 2 s to rate). Each trial was then followed by a variable inter-trial interval (ITI; 0–8 s). This rating period was included to ensure processing of the emotionality of each face and encourage introspection (Figure 1).

Behavioral data were analyzed using SPSS (Version 25.0.0; SPSS, INC.), including the use of PROCESS for mediation analyses (Hayes, 2012). Here, we chose not to use a causal steps approach to mediation, as this method has been criticized as being unnecessarily restrictive and can lead to misleading or false conclusions (e.g., concealing a significant indirect effect due to the absence of a total or direct effect, i.e., suppression; MacKinnon et al., 2000; Rucker et al., 2011). Furthermore, we did not seek to demonstrate causal effects, but rather describe relationships among traits. For these reasons, a bootstrapping method was undertaken to test for indirect effects (Preacher and Hayes, 2004).

### Neuroimaging Methods

#### Imaging Data Acquisition

All structural MRI images were acquired using a Siemens 3-T Skyra MR scanner located at the University of Louisville, School of Medicine. A 20-channel head coil was used for radiofrequency reception. Participants were given earplugs to reduce scanner noise and headphones to receive instructions. Foam padding was added to limit motion if additional room remained within the head coil, and a piece of folded tape was placed over the participant’s forehead as a reminder to remain still throughout the scan. Structural images were obtained *via* a T1-weighted magnetization-prepared rapid gradient-echo sequence (MPRAGE) in 208 sagittal slices. Imaging parameters were as follows: echo time (TE) = 2.26 ms, repetition time (TR) = 1,700 ms, flip angle = 9.0°, field of view (FoV) = 204 mm, and voxel size = 0.8 mm^3^. Scan parameters were consistent for all imaging sessions. Functional blood oxygenation level-dependent (BOLD) images were collected using gradient-echo T2*-weighted echoplanar imaging (TE = 28 ms; TR = 2,000 ms; flip angle = 79°; FoV = 204 mm; voxel size = 3.2 mm^3^; 38 interleaved slices). Slices were oriented obliquely along the AC–PC line.

### Imaging Analyses

#### Functional Analyses

Image preprocessing and data analysis were implemented using the FSL package (version 5.0.9, Analysis group, FMRIB, Oxford, UK^1^). Standard preprocessing was applied: MCFLIRT—linear slice-time correction/motion correction, optiBET—brain extraction (Lutkenhoff et al., 2014), time-series prewhitening, and high-pass filtering (0.01 Hz). Individual’s functional images were first registered to their high-resolution MPRAGE scans *via* 6-parameter linear registration, and the MPRAGE images were in turn registered to the Montreal Neurological Institute (MNI) 152 T1–2 mm template *via* a 12-parameter nonlinear registration (Andersson et al., 2007). These registrations were combined to align the functional images to the standard template. Functional images were resampled into the standard space with 2 mm isotropic voxels and were smoothed with a Gaussian kernel of 6 mm full-width at half-maximum (FWHM). ICA-AROMA (i.e., Independent Component Analysis Automatic Removal of Motion Artifact), a data-driven method to identify and remove components representing motion-related noise in fMRI data, was additionally utilized (Pruim et al., 2015). Following preprocessing, Lower-level statistics were implemented in FEAT. Using multiple regression analysis, statistical maps representing the association between the observed time-series (e.g., BOLD signal) and one or a linear combination of regressors for each subject were constructed. Regressors for the main effects were constructed by modeling each of the conditions—Fearful and Neutral—versus low-level fMRI baseline (ITI fixation), in order to create contrasts of interest: Fearful > Neutral (F > N) and Neutral > Fearful (N > F). For each regressor, a double-gamma hemodynamic response function (HRF) was convolved with an event vector starting at the stimulus onset through rating response to capture both the stimulus processing and introspective time periods in each trial (duration of 6,000 ms). Higher-level analysis was conducted using FLAME 1+2 and outlier de-weighting to combine and spatially normalize all subjects. The higher-level models employed nonparametric permutation methods through FSL’s randomize function (Nichols and Holmes, 2002). For each contrast of interest, cluster thresholding and correction for multiple comparisons were implemented through the Threshold-Free Cluster Enhancement (TFCE) method, which detects clusters of contiguous voxels without first setting an arbitrary statistical cutoff (e.g., *Z* > 2.58), and controls the family-wise error (FWE) rate at *p* < 0.05 (Smith and Nichols, 2009). Each contrast underwent 5,000 permutations. Randomise produces corrected 1-*p* maps, which were used for all figures. Figures of statistical brain maps were created using FSLview.

The present analyses primarily focused on a region of interest (ROI) approach. For ROI analyses, regions comprising a canonical socioemotional processing network were analyzed (i.e., regions supporting emotion processing and social cognition). ROIs included: bilateral TPJ, bilateral insula, mPFC, and bilateral amygdala. Convergent findings from neuroimaging and lesion studies indicate that the amygdala and insula are critical for affective reactivity and interoceptive awareness, respectively (Craig, 2002, 2009; Etkin and Wager, 2007), while the TPJ and mPFC are essential to the perception of intentionality and mental states of others, as well as cognitive empathy (Amodio and Frith, 2006; Young et al., 2010). Bilateral anatomical masks were created from FSL’s Harvard Oxford cortical atlas (insula and mPFC) and subcortical atlas (amygdala). Since this cortical atlas does not include anatomical TPJ masks, bilateral masks were manually created, using 10 mm radii surrounding the vertices at which the temporal and parietal lobes meet, at the posterior end of the Sylvian fissure for each hemisphere. The STS was additionally tested (−60, −46, 6) but yielded null results after correcting for multiple comparisons and is thus not discussed further. Secondly, exploratory whole-brain analyses were carried out for the Face Processing Task (Figure 7; Supplementary Figure S1).

**Figure 2:**
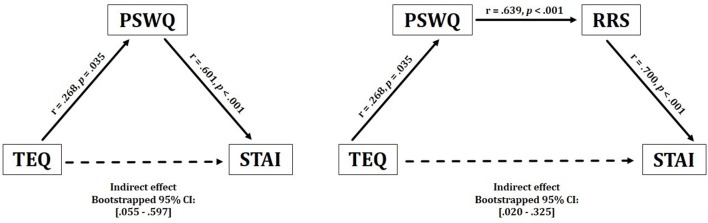
Behavioral results. Empathy demonstrated a significant positive indirect relationship with anxiety, through inconsistent mediation of worry. These effects were observed bidirectionally. Empathy also revealed a positive indirect relationship with anxiety through worry and rumination. TEQ, Toronto Empathy Questionnaire; PSWQ, Penn State Worry Questionnaire; RRS, Ruminative Responses Scale; STAI, State Trait Anxiety Inventory.

**Figure 3:**
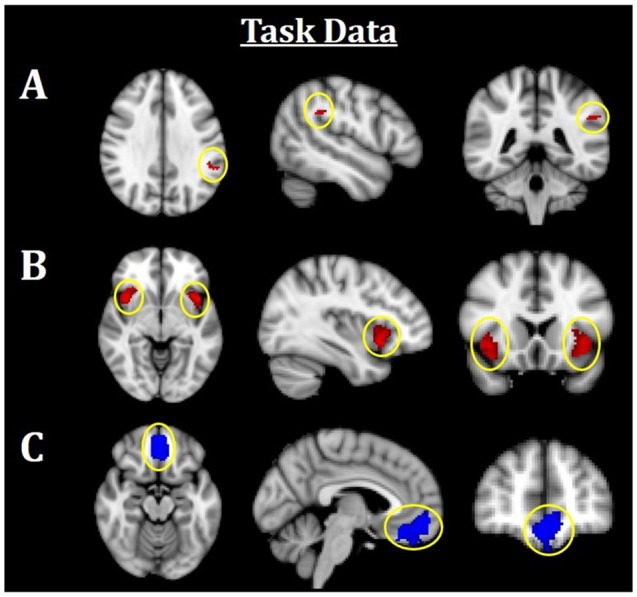
Neuroimaging task results. All results are family-wise error (FWE)-corrected, *p* < 0.05. Red indicates Fearful > Neutral (F > N) faces and blue indicates Neutral > Fearful (N > F) faces. **(A)** Greater left TPJ activation was related to F > N. **(B)** Greater bilateral insula activations related to F > N. **(C)** Increased mPFC activation was related to N > F. TPJ, temporoparietal junction; mPFC, medial prefrontal cortex.

**Figure 4:**
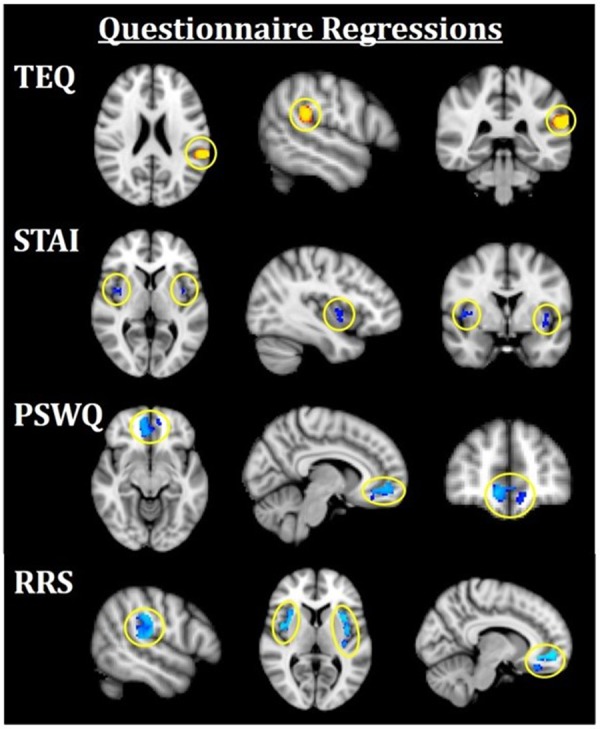
Neuroimaging results for questionnaire score regressions. All results are FWE-corrected, *p* < 0.05. Red/yellow indicates F > N faces and blue indicates N > F faces. TEQ: higher empathy related to greater activation in the left TPJ (F > N). STAI: increased anxiety related to greater bilateral insula activations (N > F). PSWQ: more worry was related to greater activation in the mPFC (N > F). RRS total: higher total rumination was related to grater activations in bilateral TPJ, bilateral insula, and mPFC (N > F). TEQ, Toronto Empathy Questionnaire; PSWQ, Penn State Worry Questionnaire; STAI, State Trait Anxiety Inventory; RRS, Ruminative Responses Scale; TPJ, temporoparietal junction; mPFC, medial prefrontal cortex.

**Figure 5:**
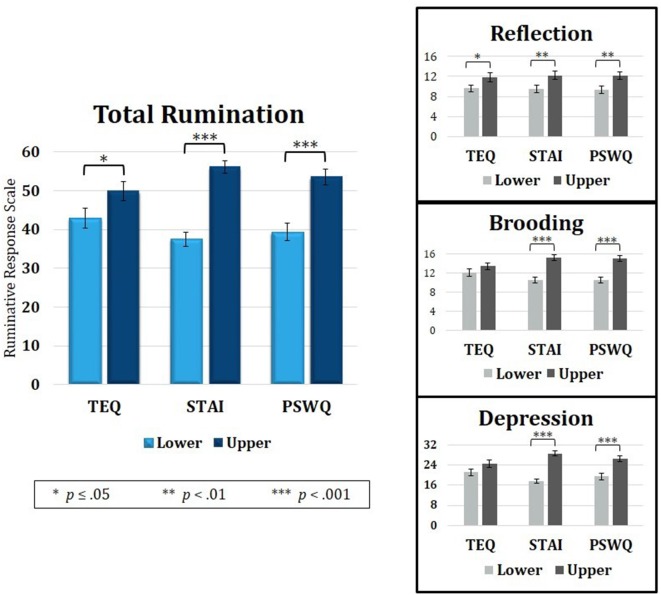
Median splits of TEQ, STAI and PSWQ showed that the Upper halves of all three questionnaires were related to higher total rumination (RRS total). Analysis of the three RRS subscales revealed that empathy, anxiety and worry were all related to higher Reflective Rumination. However, only the Upper halves of anxiety and worry were associated with higher Brooding and Depressive rumination. TEQ, toronto empathy questionnaire; STAI, State-Trait Anxiety Inventory; PSWQ, penn state worry questionnaire; RRS, Ruminative Responses Scale.

**Figure 6:**
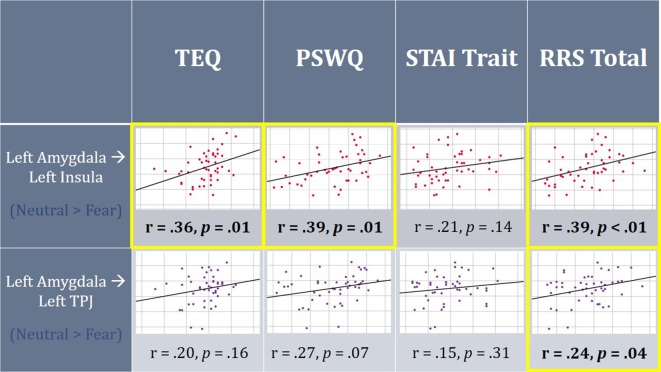
On average, individuals displayed increased functional connectivity (FC) between the left amygdala and left insula, as well as left amygdala and left TPJ for F > N faces. Higher empathy, worry and rumination was found to be positively correlated with FC between the left amygdala and left insula for N > F faces. Additionally, higher total rumination was related to increased connectivity between the left amygdala and left TPJ for N > F faces. TEQ, Toronto Empathy Questionnaire; STAI, State-Trait Anxiety Inventory; PSWQ, Penn State Worry Questionnaire; RRS, Ruminative Responses Scale; TPJ, temporoparietal junction.

**Figure 7:**
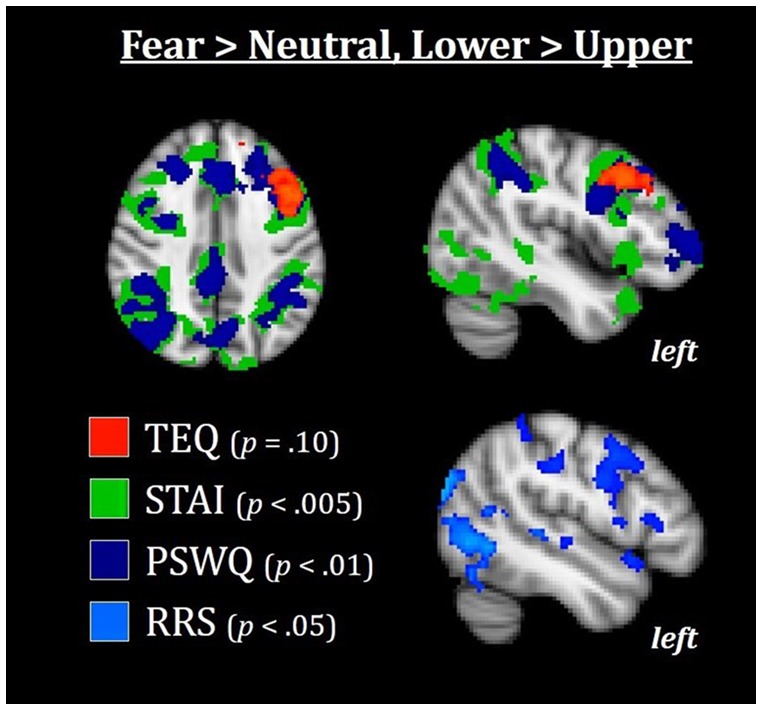
Median splits of TEQ, STAI, PSWQ, and RRS total revealed that the Lower halves of these traits were associated with enhanced activations in the frontoparietal attention network, with TEQ exhibiting one overlapping but non-significant cluster in the left dlPFC. This increased top-down activation for individuals in the Lower halves of these traits may facilitate switching between emotional and non-emotional states. All results are FWE-corrected. TEQ, Toronto Empathy Questionnaire; STAI, State-Trait Anxiety Inventory; PSWQ, Penn State Worry Questionnaire; RRS, Ruminative Responses Scale; dlPFC, dorsolateral prefrontal cortex.

#### Functional Connectivity

Seed regions for FC analyses were derived from peak z-stats of functional activation task results (peak z-stat within FWE-corrected cluster, when applicable, or peak z-stat within ROI mask in cases when no significant functional activation results were found). Seed masks were created using a 5 mm radius surrounding the peak z-stat. Seed-to-ROI FC was then performed by using the following steps. First, lower-level subject specific models (FSL’s FEAT) were run by applying high-pass filtering (100 s, and subsequently the residuals and mean functional output were added together (FSL’s res4d and mean_func). Following image preprocessing, lower-level subject-specific models were run by regressing out average time courses over ventricles, white matter and subject-space whole-brain masks (using FSL’s means). The residuals (res4d) and mean functional output (mean_func) from the FEAT output were subsequently combined to create a subject-specific preprocessed time series that was globally normalized and controlled for white matter and ventricle signals. This data was then used in a third series of lower-level subject-specific models that incorporated regressors of demeaned timeseries extracted from each seed region for each condition of interest. Thus, for each subject, we determined regions of coactivation to the specified seed within each contrast of interest, which was then combined at the group level with FLAME 1+2 mixed effects modeling. Final results were determined through paired-sample *t*-tests for each contrast of interest (F > N and N > F) using the TFCE method (FWE rate: *p* < 0.05, 5,000 permutations). Additionally, seed to whole-brain exploratory whole-brain were carried out (Supplementary Figure S2). To test whether controlling for age or sex would alter any of the reported results, both age and sex were regressed into neuroimaging task data and were not found to significantly relate to any reported regions.

#### Questionnaires

Questionnaires were incorporated into all functional analyses. First, questionnaire scores were used as regressors in higher-level ROI functional analyses in the F > N and N > F contrasts (FWE-corrected, *p* < 0.05). Additionally, regions displaying significant FC were masked using a 5 mm radius, and mean FC parameter estimates between regions were extracted for contrasts of interest and correlated with scores from each questionnaire. Finally, median splits of questionnaire scores were used for exploratory whole-brain group analyses, comparing Lower and Upper halves of each questionnaire for F > N and N > F contrasts. Areas of regional overlap between questionnaires following TFCE correction were masked using a 5 mm radius, and parameter estimated were extracted to calculate Cohen’s d effect size between the Lower and Upper halves of significant results.

## Results

### Behavioral Results

Descriptive statistics of all self-report questionnaires are shown in Table 1. Correlations of self-report questionnaires revealed that empathy was positively associated with worry (*r* = 0.30, *p* = 0.04). Worry, rumination and anxiety were found to all be highly intercorrelated (worry and rumination: *r* = 0.64, *p* < 0.001; worry and anxiety: *r* = 0.60, *p* < 0.001; rumination and anxiety: *r* = 0.70, *p* < 0.001). Contrary to hypotheses, empathy was not directly associated with anxiety (*r* = −0.04, *p* = 0.77). However, empathy positively correlated with worry (PSWQ), while worry, rumination (RRS) and anxiety (STAI) were all highly intercorrelated (Table 2).

**Table 1:** Descriptive statistics for questionnaire measures (*N* = 49).

	Min	Max	Mean	SD
TEQ	26	62	48.65	7.29
PSWQ	22	76	51.86	14.23
STAI trait	23	61	38.55	10.21
RRS total	25	76	46.59	12.61

*Note. Possible value ranges are: TEQ = 0–64, PSWQ = 16–80, STAI trait = 20–80, RRS total = 22–88. TEQ, Toronto Empathy Questionnaire; PSWQ, Penn State Worry Questionnaire; RRS, Ruminative Responses Scale; STAI, State Trait Anxiety Inventory*.

**Table 2:** Correlations between behavioral questionnaires.

	TEQ	PSWQ	RRS total	STAI trait
TEQ	1.00			
PSWQ	***r* = 0.30, *p* = 0.04**	1.00		
RRS total	*r* = 0.15, *p* = 0.30	***r* = 0.64, *p* < 0.001**	1.00	
STAI trait	*r* = −0.04, *p* = 0.77	***r* = 0.60, *p* < 0.001**	***r* = 0.70, *p* < 0.001**	1.00

*Note. Significant correlations are shown in bold. TEQ, Toronto Empathy Questionnaire; PSWQ, Penn State Worry Questionnaire; RRS, Ruminative Responses Scale; STAI, State Trait Anxiety Inventory*.

Given the lack of a direct effect between empathy anxiety, but a significant positive correlation between empathy and worry, an inconsistent mediational model was run to test for indirect effects. While classical mediation involves causal and directional relationships among variables, neither aspect is a necessary part of inconsistent mediation (i.e., suppression hypothesis). Instead, inconsistent mediation arises when the addition of a third variable actually increases the predictive validity between a predictor and outcome variable. Thus, significant indirect effects may exist even in the absence of a significant *c* (X → Y) pathway (MacKinnon et al., 2000; Zhao et al., 2010). Therefore, it was hypothesized that empathy and anxiety would be indirectly related through the process of worry.

Results from this analysis demonstrated a significant positive indirect effect between empathy and anxiety through worry, suggesting that higher empathy relates to higher worry, which in turn increases anxiety [TEQ: *p* = 0.04, PSWQ: *p* < 0.001, Bootstrapped 95% CI: (0.055–0.597)]. These effects were observed bidirectionally (STAI → PSWQ → TEQ), revealing an overall positive indirect relationship between empathy and anxiety, but only with the inclusion of worry. Furthermore, worry and rumination were included in a single model, which additionally revealed significant positive indirect effects between empathy and anxiety through both worry and rumination [TEQ: *p* = 0.03, PSWQ: *p* = 0.01, RRS: *p* < 0.001, Bootstrapped 95% CI: (0.020–0.325)]. This relationship was observed bidirectionally as well (STAI → RRS → PSWQ → TEQ), however, the positions of worry and rumination could not be interchanged as empathy only shared a direct relationship with worry (Figure 2).

### Neuroimaging Results

#### Task-Related Region of Interest Analysis

Using an established socioemotional process network (TPJ, insula, mPFC, amygdala), we first evaluated regional activations when individuals were processing fearful and neutral human faces (F > N and N > F). Results revealed that greater left TPJ activation was related to F > N faces, indicating enhanced face processing and supporting mentalizing. Greater bilateral anterior insula activation frequently associated with interoceptive awareness, was also related to F > N faces. Finally, increased mPFC activation was related to N > F faces, suggesting internal mentation, reflection or rumination (Figure 3; Table 3). No significant results were found related to amygdala. Upon further investigation, significant increased amygdala activations were observed in both main effects, in line with many studies that have shown amygdala activations for all face processing (Somerville et al., 2004; Todorov and Engell, 2008), and thus differences did not emerge in either contrast. Nevertheless, these results support that integral components of a canonical socioemotional networks were activated during this Face Processing Task.

**Table 3:** Neuroimaging task results, as shown in Figure 3.

Region		Peak *t*-statistic	*x*	*y*	*z*
Temporoparietal junction (TPJ)	L	3.98	−54	−44	32
Insula	R	4.20	40	12	−6
Insula	L	3.99	−38	16	−8
Medial prefrontal cortex (mPFC)	R/L	4.42	2	30	−22

*Coordinates in MNI Space*.

#### Questionnaire Regressions

Next, questionnaires measuring empathy, anxiety and worry were regressed into task data to investigate how these individual traits modulate processing within this socioemotional network. Higher empathy (TEQ) was found to be related to greater activation in the left TPJ for F > N faces, suggesting that higher empathy is related to enhanced face processing and mentalizing, specifically for emotional faces. Regression of STAI revealed that higher trait anxiety was related to greater bilateral insula activations for N > F faces. Although on average, individuals showed increased insula activations for F > N faces, these results demonstrate an interacting effect of anxiety, suggesting either that people higher in anxiety process the neutral faces as more negative, or exhibit prolonged interoceptive processing and emotional carry-over from the fearful faces. Similarly, higher worry (PSWQ) was found to be related to greater mPFC activation for N > F, indicating more internal mentation, and supporting the latter idea of continued processing of emotional stimuli into the neutral condition. Finally, regression with the RRS revealed that higher total rumination was associated with increased activations of bilateral TPJ, bilateral insula and mPFC for N > F faces, an amalgamation of the three results from empathy, anxiety and worry (Figure 4; Table 4).

**Table 4:** Neuroimaging results for questionnaire score regressions, as shown in Figure 4.

Region		Peak *t*-statistic	*x*	*y*	*z*
**TEQ**					
Temporoparietal junction (TPJ)	L	4.76	−58	−38	22
**STAI**					
Insula	R	3.34	46	0	2
Insula	L	3.13	−36	2	−6
**PSWQ**					
Medial prefrontal cortex (mPFC)	R/L	3.57	10	50	−8
**RRS**					
Temporoparietal junction (TPJ)	R	3.92	54	−20	16
Temporoparietal junction (TPJ)	L	4.32	−54	−38	34
Insula	R	4.04	32	16	−2
Insula	L	4.87	−34	12	10
Medial prefrontal cortex (mPFC)	R/L	3.88	12	52	−6

*Coordinates in MNI Space*.

Because rumination revealed overlapping neural correlates with empathy, anxiety and worry, the behavioral data was revisited to assess rumination as a connecting point. Median splits of TEQ, STAI, and PSWQ were performed, and independent samples *t*-tests were calculated on degree of rumination between the Lower and Upper half groups of each questionnaire. Results showed that individuals in the Upper halves of empathy, anxiety and worry also had higher total rumination on average (TEQ: *t*_(47)_ = 1.98, *p* = 0.05; STAI: *t*_(47)_ = 7.59, *p* < 0.001; PSWQ: *t*_(47)_ = 4.75, *p* < 0.001). Rumination scores were then further divided into the three subscales—Reflection, Brooding and Depressive Rumination. Within the Reflection subscale, individuals in the Upper halves of empathy, anxiety and worry all had higher Reflective Rumination (TEQ: *t*_(47)_ = 2.00, *p* = 0.05; STAI: *t*_(47)_ = 2.54, *p* = 0.01; PSWQ: *t*_(47)_ = 2.56, *p* = 0.01). However, for the Brooding (TEQ: *t*_(47)_ = 1.18, *p* = 0.25; STAI: *t*_(47)_ = 5.34, *p* < 0.001; PSWQ: *t*_(47)_ = 4.93, *p* < 0.001) and Depression subscales (TEQ: *t*_(47)_ = 1.73, *p* = 0.09; STAI: *t*_(47)_ = 9.10, *p* < 0.001; PSWQ: *t*_(47)_ = 4.75, *p* < 0.001), only individuals in the Upper halves of anxiety and worry had significantly higher Brooding and Depressive Rumination (Figure 5).

#### Functional Connectivity

Next, FC was performed between seeds and all regions comprising the socioemotional network. Significant increased FC was observed between the left amygdala and left insula (peak *t*-statistic = 4.72; −38, −6, −4), as well as the left amygdala and left TPJ (peak *t-statistic* = 4.37; −52, −30, 28) for F > N faces. This demonstrates that on average, individuals displayed increased communication between these regions when viewing emotional faces compared to neutral ones. However, when relating these findings to questionnaire scores, higher empathy, worry and total rumination were all related to increased connectivity between the left amygdala and left insula in the N > F contrast (TEQ: *r* = 0.36, *p* = 0.01; PSWQ: *r* = 0.39, *p* = 0.01; RRS: *r* = 0.39, *p* = 0.01), suggesting not only enhanced communication between these regions in response to emotional faces, but sustained connectivity into the neutral condition for people higher on these traits. Similarly, increased total rumination was related to greater connectivity between the left amygdala and left TPJ in N > F (RRS: *r* = 0.24, *p* = 0.04; Figure 6).

#### Whole-Brain Analysis

Finally, we wanted to explore whether increased bottom-up processing or decreased top-down control seemed to be driving this putative emotional carry-over from Fearful to Neutral trials. In other words, could we find either: (1) evidence for increased bottom-up processing in individuals high on these measures, suggesting greater sensitivity to social and emotional information; and/or (2) decreased top-down attentional control in individuals high on these measures, suggesting more difficulty in switching between emotional and non-emotional states? To test this, we performed an exploratory whole-brain analysis using the median split groups (with the addition of median split RRS total), comparing the Lower and Upper half groups of each questionnaire in the F > N and N > F contrasts. Results showed that in F > N, the Lower half groups were all associated with increased activations in frontoparietal attentional networks, with strong similarities and regional overlap between anxiety, worry and rumination [average Cohen’s d for Lower>Upper across questionnaires for left dorsolateral PFC (dlPFC) = 1.12, and for left intraparietal sulcus (IPS) = 0.98]. For empathy, one overlapping but non-significant cluster in the left dlPFC was observed (FWE-corrected, *p* = 0.10). No significant results were found for F > N, Upper > Lower. Therefore, individuals on the Upper end of any or all of these measures appear to exhibit decreased top-down attentional control during the F > N contrast, leading to both enhanced processing of emotional stimuli and prolonged processing into neutral situations (Figure 7).

## Discussion

The present study aimed to investigate the convergent neural correlates that may underlie a relationship between empathy and anxiety. We used an emotional Face Processing Task shown to reliably elicit activation from brain regions in a canonical socioemotional network, self-report questionnaires measuring empathy, anxiety and internally generated thought (i.e., worry and rumination), to examine how these traits were related to functional activation within, and communication between, regions comprising a socioemotional processing network. Our results show that empathy, anxiety and worry each engaged a different component of this socioemotional network, while rumination related to increased activations across all cortical regions in the network. Furthermore, higher empathy, worry and rumination were all associated with increased bottom-up connectivity, while anxiety was related to worry and rumination through decreased top-down attentional control. Taken together, our data demonstrate that in the context of socioemotional processing, empathy shares an indirect relationship with anxiety through the ruminative tendencies of worry.

### Behavioral Associations

Behaviorally, our results showed a positive correlation between empathy and worry, as measured by the TEQ and PSWQ, respectively. Empathy is closely tied to perspective-taking and mentalizing, which requires using one’s own mental state to gain insight into another’s thoughts or feelings. Comparatively, worry tends to be a future-oriented state that focuses on potential threats, but also represents an attempt at mental problem-solving (Borkovec et al., 1983). Thus, this positive relationship between empathy and worry suggests that predispositions towards empathic arousal and perspective-taking may be natural to engage in problem-solving simulations on behalf of other individuals. Additionally, we observed a significant indirect relationship between empathy and anxiety through the process of worry, as well as through worry and rumination. While rumination shares many similarities to worry, it has been suggested that rumination is a process of “compulsively focusing attention on the symptoms of one’s distress, and on its possible causes and consequences, as opposed to its solutions” (Nolen-Hoeksema, 1998). This idea both supports the observation that rumination is most closely related to anxiety, and provides a scaffold for how empathy relates to worry, which in turn relates to rumination, and thus anxiety.

### Relationships to Functional Activation

Questionnaire regressions further revealed that each of these processes—empathy, anxiety and worry—related to increased activations within different cortical regions comprising a socioemotional network. First, empathy was found to relate to increased activation in the left TPJ for F > N faces. The TPJ is known to be a multimodal association area that integrates input from visual, auditory, somatosensory and limbic areas, and has reciprocal connection to the PFC, making it a central locus for processing multisensory information and cognitive aspects related to the self (Decety and Lamm, 2006). In concert with the posterior STS, the TPJ is also thought to be specialized for processing faces and eye gaze (Blakemore, 2008), and thus aids in mentalizing (Bernhardt and Singer, 2012). While the right TPJ is more commonly implicated in empathetic responses, studies have shown involvement of bilateral TPJ when participants read stories about character’s mental states or false beliefs (Young et al., 2010), and specifically the left TPJ when participants imitated others (Decety and Lamm, 2006). Taken together, this suggests that increased empathy may be related to enhanced face processing and mentalizing during the current study, particularly for emotional faces.

**Figure 8:**
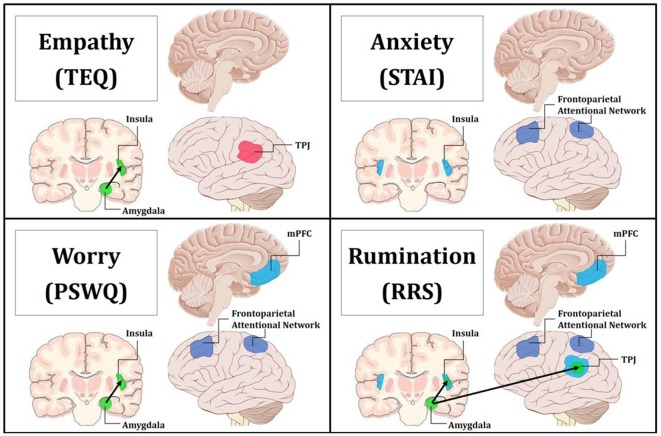
Summary figure of neuroimaging findings related to each trait. Behaviorally and neurally, empathy shared direct relationships with worry and rumination. Additionally, anxiety exhibited behavioral relationships and common neural correlates with worry and rumination. Empathy was found to only share a significant indirect behavioral relationship with anxiety through worry, and this is reflected in the convergent neural correlates of worry and rumination. Pink indicates increased regional activations for F > N. Blue indicates increased regional activations for N > F. Green with black arrows denotes increased FC for N > F. Purple represents decreased top-down attentional control for F > N when comparing the Upper > Lower halves of questionnaires. Top Left: empathy was positively related to increased activation in the left TPJ for F > N, and increased FC between the left amygdala and insula for N > F. Top Right: anxiety was related to increased activations in bilateral insula for N > F, and decreased activations in the frontoparietal attentional network for Upper > Lower. Bottom Left: worry was related to increased activation in the mPFC for N > F, increased FC between the left amygdala and insula for N > F, and decreased activations in the frontoparietal attentional network for Upper > Lower. Bottom Right: rumination was related to increased activation in the mPFC, bilateral insula and bilateral TPJ for N > F, increased FC between the left amygdala and insula as well as the left amygdala and TPJ for N > F, and decreased activations in the frontoparietal attentional network for Upper > Lower.

Regression of STAI trait revealed increased activations in bilateral anterior insula for Neutral trials, and similarly, higher worry was related to increased mPFC activation for Neutral trials, indicating that these traits are related to prolonged processing of emotional stimuli. Research suggests that the insula plays an important role in vicariously sharing emotions (Bernhardt and Singer, 2012), as well as representing and integrating interoceptive and affective states (Craig, 2002, 2009). In fact, in one study looking at high and low degrees of alexithymia, the greater the individual’s deficits in understanding their own emotions, the less insula activation they showed while empathizing with others in pain (Bird et al., 2010). Therefore, these increased activations in the anterior insula supports the notion that trait anxiety is related to increased emotional sensitivity and interoceptive awareness. Furthermore, the mPFC has been linked to many aspects of social cognition, including monitoring one’s own emotional state (Dvash and Shamay-Tsoory, 2014), understanding emotional states of others (Amodio and Frith, 2006), and internal mentation (Andrews-Hanna, 2012). Depressed participants show significantly greater activation in the mPFC than controls during experimentally induced rumination (Cooney et al., 2010), and individuals with generalized anxiety show sustained activation of the mPFC into resting epochs, which correlates with PSWQ scores (Paulesu et al., 2010). Together, this suggests that higher anxiety may promote continued interoceptive awareness into Neutral trials, while higher worry may play a role in cognitively processing those feelings (i.e., interoceptive processing and introspection). Finally, regression of RRS total showed that rumination was related to increased activations in bilateral TPJ, bilateral anterior insula, and mPFC for Neutral trials, suggesting that rumination is globally related to prolonged processing of emotional information, and moreover, alludes to relationships with empathy, anxiety and worry *via* overlapping neural correlates (summary in Figure 8).

### Empathy Relates to Reflection, Worry to Depressive Brooding

Upon revisiting the behavioral data to further assess rumination as a connecting point between empathy and anxiety, median splits revealed that higher empathy, anxiety and worry were all related to higher total rumination. However, when assessing the rumination subscales, individuals in the Lower and Upper halves of empathy showed no differences on Brooding and Depression, suggesting that empathy is most closely tied to Reflective Rumination. In line with this finding, research indicates that self-reflection is positively correlated with perspective-taking and empathic concern (Joireman et al., 2002). On the other hand, the Lower and Upper halves of anxiety and worry showed significant differences in levels of Brooding and Depressive Rumination. Evidence suggests that the Brooding subscale is more pathological than Reflection, and Depressive Rumination is known to consist of items that overlap with measures of depression symptomatology (Treynor et al., 2003; Watkins and Moulds, 2009). Furthermore, one study showed that rumination (measured separately from self-reflection) is in fact negatively related to perspective-taking, and is instead associated with personal distress (Joireman et al., 2002). Both worry and rumination have been described as unproductive and repetitive thought processes (Segerstrom et al., 2000) that exacerbate and prolong negative affect (Nolen-Hoeksema et al., 2008), and it is believed that this repetitive negative thinking is what increases vulnerability to multiple anxiety and depressive disorders (McEvoy et al., 2013). Therefore, while empathy shares a similar style of repetitive thinking and continued processing of emotional information through reflection, only worry and anxiety demonstrated a relationship with ruminative subscales associated with repetitive *negative* thinking typical of many clinical mood disorders. Because the relationship between empathy and anxiety was only found indirectly through worry, or worry and total rumination, this suggests that the tendency towards negative affect in worry and rumination may be a critical component necessary to link empathy to anxiety. Thus, empathy may be directly tied to worry through metalizing and simulations of situational outcomes, but the indirect relationship to anxiety seems to rely on persistent negative affect induced by repetitive negative thinking found in the ruminative tendencies of worry.

### Relationships to Functional Connectivity

We next evaluated region-to-region FC within this canonical socioemotional network for the Face Processing Task, and assessed the modulatory role these individual differences play. Interestingly, although no functional activation differences emerged in the amygdala, significant increased FC was observed between the left amygdala and left insula, as well as the left amygdala and left TPJ during Fear trials. When these findings were then related to our self-report measures, we found that higher empathy, worry and rumination were all related to increased connectivity between the amygdala and insula during Neutral trials, while rumination was additionally related to connectivity between the amygdala and TPJ for Neutral trials. Rumination of all types has been shown to relate to increased and sustained amygdala reactivity (Mandell et al., 2014), lasting throughout subsequent non-emotional trials (Siegle et al., 2002), and our results add that rumination may also be related to altered amygdala connectivity. Additionally, in support of our findings connecting empathy, worry and rumination through increased bottom-up connectivity, studies on autism spectrum disorders (ASDs), widely thought to be related to deficits in empathy, show decreased FC between the amygdala and insula (von dem Hagen et al., 2013), while resting state fMRI accounts report increased FC between these regions in relationship to state anxiety (Baur et al., 2013). Of interest, these FC results add to a growing list of curiously left-lateralized findings given the emotional nature of the task. However, worry, rumination and mentalizing are largely verbal or linguistic by nature (Fresco et al., 2002), and furthermore, participants were instructed to actively view and evaluate each face, hence promoting a more motivated “approach” response (Harmon-Jones et al., 2006), both of which are predominantly left-lateralized. Nevertheless, collectively with the results from regional functional activation, these findings reinforce the assertion of enhanced and prolonged emotional processing in association with these traits and outline a common neural mechanism linking empathy with worry/rumination (summary in Figure 8).

### Reduced Top-Down Attention Drives Prolonged Emotion Processing

Finally, we pursued an exploratory whole-brain analysis to assess whether these neural commonalities were being driven by increased bottom-up processing, and/or decreased top-down control. Compared to the Upper halves, the Lower halves of anxiety, worry and rumination, but not empathy, were all associated with increased activations in the frontoparietal attentional network for fearful than neutral faces. This implies that in a naturalistic socioemotional setting, higher scores on these traits are all related to decreased top-down attentional control. Decreased activation in the dlPFC is thought to underlie disinhibition, allowing for sustained engagement of emotional-processing structures (Siegle et al., 2002). In support of this notion, higher brooding tendencies are associated with more errors when attempting to inhibit negative information (Vanderhasselt et al., 2011). Notably, these results demonstrate a common neural mechanism between anxiety, worry and rumination, but a *dissociation* from empathy, providing the indirect link between empathy and anxiety through worry that was previously observed behaviorally (summary in Figure 8).

### Limitations

It should be noted that all measures of empathy, anxiety, worry and rumination were collected through self-report questionnaires, which have their limitations. In light of this, future studies should continue to investigate the relationship between empathy and anxiety using performance measures, or other tasks using a broader range of stimuli types that cover more diverse emotional states. Future research should additionally extend these findings by untangling the relationship between anxiety, worry and rumination with cognitive and affective empathy, which display both behavioral and neurologically distinct mechanisms (Preckel et al., 2018). Furthermore, the present study was conducted as part of a much larger investigation on emotion regulation in young adults, and as such, only one questionnaire for each construct was collected. While a multi-method multi-trait approach would be ideal, we have attempted to demonstrate that the utilized questionnaires have strong internal reliability and discriminant validity in measuring their putative constructs (Supplementary Tables S1–S6). Additionally, all of our participants were considered psychologically healthy, and while the measured empathy/anxiety/worry/rumination scores covered a broad range (Table 1), it would be worthwhile to explore whether individuals with clinical disorders show corresponding results. Previous literature has documented that many psychological disorders such as ASD (Baron-Cohen et al., 2005) and antisocial personality disorder (Blair, 2001) demonstrate decreased empathy and impaired social functioning, however other studies have also provided evidence that social information processing is often impaired in individuals with anxiety disorders (Luebbe et al., 2010). It is possible that by excluding the extreme ends of each spectrum, our observed linear relationships may not be capturing the whole picture. Finally, although our sample size was ample with nearly equal numbers of males and females, looking at gender differences was beyond the scope of this article. However, because there are known gender differences in empathy (Schulte-Rüther et al., 2008), emotional processing (Brody and Hall, 1993; McRae et al., 2008), and prevalence of psychiatric disorders (Nolen-Hoeksema, 2001; McLean et al., 2011), future studies should explore how these neural mechanisms may vary by gender.

### Summary and Conclusions

In conclusion, the present study sought to investigate neural commonalities that may support correspondence between trait empathy and anxiety in a healthy population. While a direct behavioral relationship was not observed between these traits, the results revealed indirect links between empathy and anxiety through the mediation of worry, and shared associations with higher self-reflection and ruminative thinking style. These findings, first seen behaviorally, were echoed through convergent neural correlates found in worry and rumination (Figure 8). First, empathy alone was related to worry and ruminative thinking through increased bottom-up communication of emotional processing regions (i.e., FC between the amygdala and insula). Further, results showed that worry and rumination shared commonalities with anxiety through decreased activations in the frontoparietal attentional network. Together this suggests that the enhanced and prolonged bottom-up processing of emotional information seen in empathy, in combination with the decreased top-down attentional control and repetitive negative thinking central to worry and rumination, may result in higher general anxiety in socioemotional encounters.

## Author Contributions

LK, TS, NF and BD were involved in designing the study. LK and TS were involved in data acquisition. LK and BD were involved in analysis and all authors were involved in the interpretation of the data. LK drafted the article. TS, NF and BD critically revised the manuscript. All authors approved the final version of the manuscript.

## Conflict of Interest Statement

The authors declare that the research was conducted in the absence of any commercial or financial relationships that could be construed as a potential conflict of interest.

## Footnotes

^1^http://www.fmrib.ox.ac.uk/fsl/
