# Supplementary material for: Convergent Neural Correlates of Empathy and Anxiety During Socioemotional Processing
Source: Front Hum Neurosci. 2019 Mar 19;13:94. doi: 10.3389/fnhum.2019.00094 (PMC6438321; doi:10.3389/fnhum.2019.00094)
Supplement: Supplementary file 1 [file Data_Sheet_1.docx]

**Supplementary Material**

In addition to exploring functional activation within and functional connectivity between our regions of interest, we also explored whole brain results for the Face Processing Task (**Supplementary Figure 1; Supplementary Figure 2**).


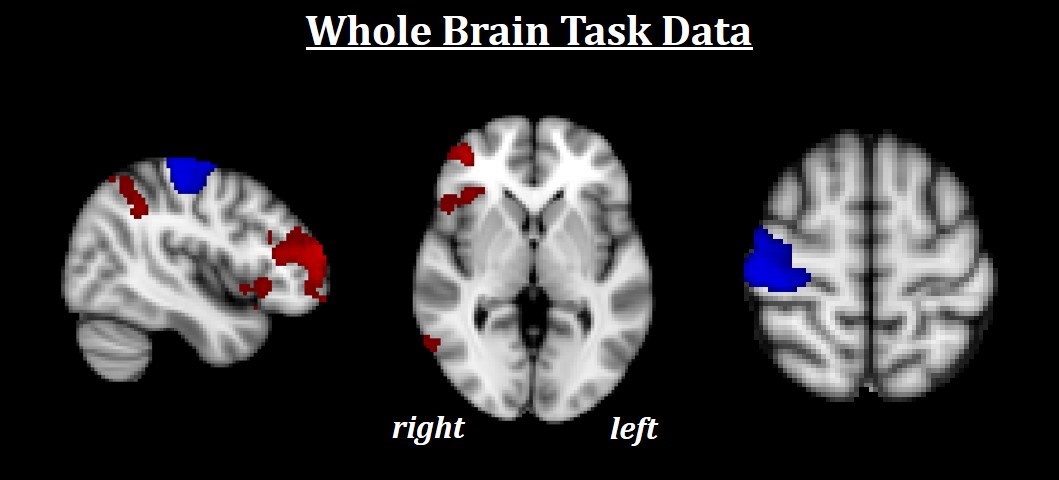


**Supplementary Figure 1:** *Whole brain results for the Face Processing Task. Red indicates increased regional activations for Fearful>Neutral (F>N). Blue indicates increased regional activations for Neutral>Fearful (N>F). All results are FWE-corrected, p < .05. During F>N, increased activations were found in the right anterior middle frontal gyrus (MFG), right inferior parietal lobule (IPL), and right anterior insula. N>F was found to be associated with increased activity in the right somatomotor cortex, likely reflecting the consistently low affective ratings (1 & 2) to neutral faces given by the left hand.*


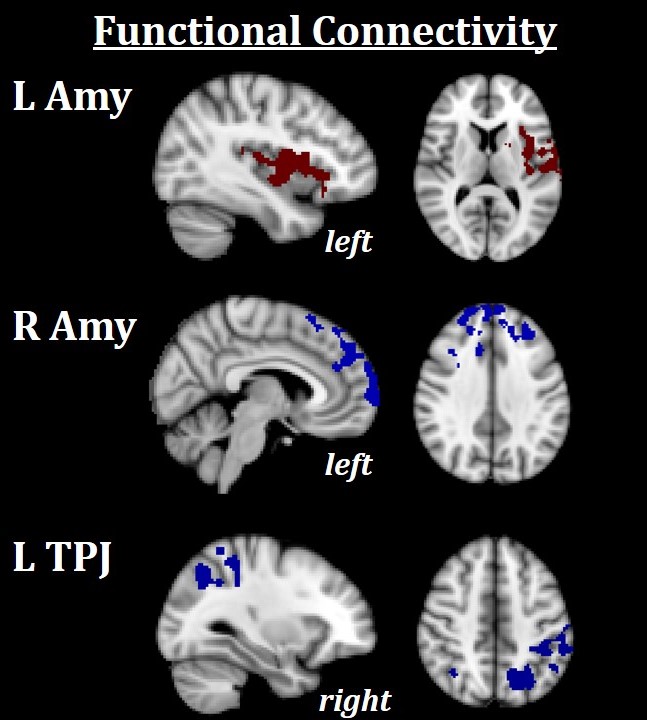


**Supplementary Figure 2:** *Seed to whole brain functional connectivity results. Red indicates increased functional connectivity for Fearful>Neutral (F>N). Blue indicates increased functional connectivity for Neutral>Fearful (N>F). All results are FWE-corrected, p < .05. Increased functional connectivity between the left amygdala and left insula was found for F>N, as also shown in the main text. Increased functional connectivity between the right amygdala and medial frontal cortex was found for N>F. Finally, increased connectivity was found between the left TPJ and predominantly right lateralized superior and inferior parietal cortex.*

*L Amy = left amygdala; R Amy = right amygdala; L TPJ = left temporoparietal junction*

Since many of these constructs are closely related (i.e. worry, rumination and anxiety), two additional analyses were conducted to increase confidence that each questionnaire uniquely measured its putative construct. First, Cronbach’s alpha was calculated for each questionnaire to measure internal reliability. Generally, an alpha value of > .7 is considered sound, and the resulting alpha values for this sample were as follows: TEQ: α = .867; PSWQ: α = .941; RRS Total: α = .915; STAI Trait: α = .909. Secondly, a discriminant validity analysis was conducted to provide evidence that these questionnaires were able to uniquely measure each of their putative constructs. To test this, Principal Axis Factoring with an oblique rotation (direct oblimin) was performed for each pair of questionnaires. Factors were retained if the Eigenvalue >2 and a cross-check with scree plot deflections supported the number of resultant factors. Factor loading cutoff was set at .4, which is considered substantial (Field, 2013). The pattern matrices were then evaluated for their ability to distinguish between questionnaires, through visual assessment of whether items from separate questionnaires primarily loaded on unique components, as coefficients of a pattern matrix represent unique individual investment of a variable to that factor. Average variance extracted (AVE) was then calculated for each factor, and discriminant validity was concluded if all AVE estimates within each factor were greater than the shared variance estimate (i.e. squared correlation) between factors (Farrell and Rudd, 2009).

The results for items from worry (PSWQ) and rumination (RRS) are shown in Supplementary Tables 1 and 2. The analysis yielded a four-factor solution. An examination of the Kaiser-Meyer Olkin measure of sampling adequacy suggested that the sample was factorable (KMO = .708) and Bartlett’s test of sphericity was significant (*p* < .001). Fifteen of the sixteen items in the PSWQ loaded onto Factor 1. It is clear from Table 1 that this factor solely relates to the PSWQ and was thus labelled “Worry”. Seven items from the RRS loaded onto the second factor. These seven items were a combination of the Brooding and Depressive scales but contained many items that related to thinking about sadness or other negative emotions and so was labelled “Emotional Rumination”. Five items from the RRS loaded onto the third component, which are the five items that comprise the Reflection subscale and so accordingly, this factor was labelled “Reflection”. Finally, seven items from the RRS loaded onto the fourth component, again comprised of items from the Brooding and Depressive subscales. However, many of these items relate to worries about difficulty concentrating, getting motivated or doing one’s job well, and so was labelled “Incapacitated Rumination”. For each of these components, AVE and composite reliability were calculated. Composite reliability is used as a check of internal consistency, and should be greater than the benchmark of .7 to be considered adequate. Worry: AVE = .448, CR = .923; Emotional Rumination: AVE = .413, CR = .823; Reflection: AVE = .479, CR = .818; Incapacitated Rumination: AVE = .379, CR = .807. Finally, all AVE values were compared to the squared correlations between factors (Supplementary Table 2). Since worry and rumination loaded cleanly onto discrete components, and AVE within all components was greater that shared variance between components, it was concluded that the PSWQ and RRS had discriminant validity.

**Supplementary Table 1**: Pattern Matrix component loadings for worry and rumination

| **Component** | **1** | **2** | **3** | **4** |
| --- | --- | --- | --- | --- |
| PSWQ 1 |  |  |  |  |
| PSWQ 2 | 0.757 |  |  |  |
| PSWQ 3 | 0.782 |  |  |  |
| PSWQ 4 | 0.432 |  |  |  |
| PSWQ 5 | 0.760 |  |  |  |
| PSWQ 6 | 0.649 |  |  |  |
| PSWQ 7 | 0.652 |  |  |  |
| PSWQ 8 | 0.662 |  |  |  |
| PSWQ 9 | 0.695 |  |  |  |
| PSWQ 10 | 0.579 |  |  |  |
| PSWQ 11 | 0.771 |  |  |  |
| PSWQ 12 | 0.570 |  |  |  |
| PSWQ 13 | 0.543 |  |  |  |
| PSWQ 14 | 0.718 |  |  |  |
| PSWQ 15 | 0.761 |  |  |  |
| PSWQ 16 | 0.603 |  |  |  |
| RRS 1 |  | 0.824 |  |  |
| RRS 2 |  |  |  | 0.730 |
| RRS 3 |  |  |  | 0.612 |
| RRS 4 |  |  |  | 0.577 |
| RRS 5 |  |  |  |  |
| RRS 6 |  |  |  | 0.545 |
| RRS 7 |  |  | 0.640 |  |
| RRS 8 |  | 0.500 |  |  |
| RRS 9 |  |  |  | 0.619 |
| RRS 10 |  |  |  |  |
| RRS 11 |  |  | 0.758 |  |
| RRS 12 |  |  | 0.597 |  |
| RRS 13 |  |  |  |  |
| RRS 14 |  |  |  | 0.728 |
| RRS 15 |  |  |  | 0.451 |
| RRS 16 |  | 0.528 |  |  |
| RRS 17 |  | 0.827 |  |  |
| RRS 18 |  | 0.718 |  |  |
| RRS 19 |  |  |  |  |
| RRS 20 |  | 0.411 | 0.566 |  |
| RRS 21 |  |  | 0.858 |  |
| RRS 22 |  | 0.564 |  |  |
| Percentage of total variance | 37.06 | 9.82 | 7.04 | 5.88 |

**Supplementary Table 2**: Factor Correlation Matrix

| **Component** | **1** | **2** | **3** | **4** |
| --- | --- | --- | --- | --- |
| **1** | 1.00 | .258 | .274 | .307 |
| **2** | .258 | 1.00 | .184 | .388 |
| **3** | .274 | .184 | 1.00 | .234 |
| **4** | .307 | .388 | .234 | 1.00 |

Next, items from worry (PSWQ) and anxiety (STAI) were assessed. Results are shown in Supplementary Tables 3 and 4. KMO = .670, Bartlett’s test of sphericity was significant (*p* < .001). The analysis yielded a three-factor solution. Again, fifteen of the sixteen items from the PSWQ loaded onto Factor 1, “Worry”. The remaining items from the STAI were split between Factor 2 and Factor 3. Factor 2 included more items relating to physical feelings associated with being anxious such as feeling tense and jittery and was labeled “Anxious arousal”, while Factor 3 included more items surrounding the emotional and cognitive aspects of anxiety and was called “Anxious Apprehension”. AVEs and CRs were as follows: Worry: AVE = .480, CR = .935; Anxious Arousal: AVE = .529, CR = .897; Anxious Apprehension: AVE = .340, CR = .818. Again, because worry and anxiety loaded cleanly onto discrete components, and AVE within all components was greater that shared variance between components, it was concluded that the PSWQ and STAI trait had discriminant validity.

**Supplementary Table 3**: Pattern Matrix component loadings for worry and anxiety

| **Component** | **1** | **2** | **3** |
| --- | --- | --- | --- |
| PSWQ 1 |  |  |  |
| PSWQ 2 | 0.674 |  |  |
| PSWQ 3 | 0.678 |  |  |
| PSWQ 4 | 0.514 |  |  |
| PSWQ 5 | 0.755 |  |  |
| PSWQ 6 | 0.756 |  |  |
| PSWQ 7 | 0.666 |  |  |
| PSWQ 8 | 0.793 |  |  |
| PSWQ 9 | 0.736 |  |  |
| PSWQ 10 | 0.649 |  |  |
| PSWQ 11 | 0.613 |  |  |
| PSWQ 12 | 0.721 |  |  |
| PSWQ 13 | 0.592 |  |  |
| PSWQ 14 | 0.803 |  |  |
| PSWQ 15 | 0.806 |  |  |
| PSWQ 16 | 0.770 |  |  |
| STAI 1 |  | 0.881 |  |
| STAI 2 | 0.428 |  |  |
| STAI 3 |  | 0.477 |  |
| STAI 4 |  |  | 0.683 |
| STAI 5 |  |  | 0.747 |
| STAI 6 |  | 0.585 |  |
| STAI 7 |  | 0.636 |  |
| STAI 8 |  |  | 0.527 |
| STAI 9 |  |  | 0.480 |
| STAI 10 |  | 0.832 |  |
| STAI 11 |  |  | 0.441 |
| STAI 12 |  |  | 0.524 |
| STAI 13 |  | 0.710 |  |
| STAI 14 |  |  |  |
| STAI 15 |  |  | 0.469 |
| STAI 16 |  | 0.791 |  |
| STAI 17 |  |  | 0.643 |
| STAI 18 |  |  | 0.652 |
| STAI 19 |  | 0.812 |  |
| STAI 20 |  |  |  |
| Percentage of total variance | 36.89 | 10.90 | 6.85 |

**Supplementary Table 4**: Factor Correlation Matrix

| **Component** | **1** | **2** | **3** |
| --- | --- | --- | --- |
| **1** | 1.00 | .299 | .469 |
| **2** | .299 | 1.00 | .374 |
| **3** | .469 | .374 | 1.00 |

Finally, rumination (RRS) and anxiety (STAI) were assessed. Results are shown in Supplementary Tables 5 and 6. The analysis yielded a five-factor solution. KMO = .591, Bartlett’s test of sphericity was significant (*p* < .001). This analysis was the only to reveal a factor that contained multiple items from both questionnaires. Six items from rumination and three items from anxiety loaded onto Factor 1, which was labelled “Anxious Rumination”. Eight items from anxiety negatively loaded on Factor 2, called “Anxiety Absent”. Again, the five items from the Reflection subscale comprised Factor 3, “Reflection”. Seven items from rumination negatively loaded onto Factor 4, “Rumination Absent”. Finally, seven items from anxiety loaded onto Factor 5, which was labelled “Anxiety Present”. AVEs and CRs were calculated for each component. Anxious Rumination: AVE = .375, CR = .840; Anxiety Absent: AVE = .469, CR = .885; Reflection: AVE = .463, CR = .809; Rumination Absent: AVE = .390, CR = .832, Anxiety Present: AVE = .285, CR = .729. The AVE within all components exceeded the squared correlations between components, indicating discriminant validity between components. However, this analysis was the only to yield one factor composed of multiple items from each questionnaire (Factor 1: “Anxious Rumination”), suggesting that rumination and anxiety share the most overlap (and did in fact share the strongest bivariate correlation). Still, two factors were found to be unique to rumination, while an additional two factors were unique to anxiety, suggesting that aspects of these two constructs can still be dissociated.

**Supplementary Table 5**: Pattern Matrix component loadings for rumination and anxiety

| **Component** | **1** | **2** | **3** | **4** | **5** |
| --- | --- | --- | --- | --- | --- |
| RRS 1 | 0.686 |  |  |  |  |
| RRS 2 |  |  |  | -0.746 |  |
| RRS 3 |  |  |  | -0.713 |  |
| RRS 4 |  |  |  | -0.555 |  |
| RRS 5 |  |  |  |  |  |
| RRS 6 |  |  |  | -0.532 |  |
| RRS 7 |  |  | 0.582 |  |  |
| RRS 8 |  | -0.648 |  |  |  |
| RRS 9 |  |  |  | -0.688 |  |
| RRS 10 |  |  |  |  |  |
| RRS 11 |  |  | 0.748 |  |  |
| RRS 12 |  |  | 0.678 |  |  |
| RRS 13 |  |  |  |  |  |
| RRS 14 |  |  |  | -0.744 |  |
| RRS 15 | 0.529 |  |  |  |  |
| RRS 16 | 0.710 |  |  |  |  |
| RRS 17 | 0.524 |  |  |  |  |
| RRS 18 | 0.592 |  |  |  |  |
| RRS 19 |  |  |  | -0.517 |  |
| RRS 20 |  |  | 0.554 |  |  |
| RRS 21 |  |  | 0.808 |  |  |
| RRS 22 | 0.522 |  |  |  |  |
| STAI 1 |  | -0.931 |  |  |  |
| STAI 2 |  |  |  | -0.413 |  |
| STAI 3 |  | -0.467 |  |  |  |
| STAI 4 | 0.512 |  |  |  |  |
| STAI 5 | 0.592 |  |  |  |  |
| STAI 6 |  | -0.552 |  |  |  |
| STAI 7 |  | -0.579 |  |  | 0.430 |
| STAI 8 |  |  |  |  |  |
| STAI 9 |  |  |  |  | 0.548 |
| STAI 10 |  | -0.803 |  |  |  |
| STAI 11 |  |  |  |  | 0.552 |
| STAI 12 |  |  |  |  | 0.454 |
| STAI 13 |  | -0.649 |  |  |  |
| STAI 14 |  |  |  |  |  |
| STAI 15 | 0.779 |  |  |  |  |
| STAI 16 |  | -0.740 |  |  |  |
| STAI 17 |  |  |  |  | 0.525 |
| STAI 18 |  |  |  |  | 0.745 |
| STAI 19 |  | -0.680 |  |  |  |
| STAI 20 |  |  |  |  | 0.416 |
| Percentage of total variance | 32.86 | 8.27 | 7.19 | 6.11 | 5.13 |

**Supplementary Table 6**: Factor Correlation Matrix

| **Component** | **1** | **2** | **3** | **4** | **5** |
| --- | --- | --- | --- | --- | --- |
| **1** | 1.00 | -.325 | .217 | -.368 | .313 |
| **2** | -.325 | 1.00 | -.151 | .275 | -.206 |
| **3** | .217 | -.151 | 1.00 | -.177 | .039 |
| **4** | -.368 | .275 | -.177 | 1.00 | -.251 |
| **5** | .313 | -.206 | .039 | -.251 | 1.00 |
